# Symptom Clusters in Brazilian Women With Stage I and Stage III Nonmetastatic Breast Cancer: A Cross‐Sectional Study

**DOI:** 10.1155/tbj/5427340

**Published:** 2026-03-01

**Authors:** Luís Carlos Lopes-Júnior, Etreo Junior Carneiro da Silva Minarini, Raphael Manhães Pessanha, Luiz Cláudio Barreto Silva Neto, Naira Santos D’Agostini, Jonathan Grassi, Karla Anacleto Vasconcellos, Roberto Júnio Gomes Silva, Leticia Batista de Azevedo, Livia Machado Giacomin, Oscar Geovanny Enriquez-Martinez, Wesley Rocha Grippa

**Affiliations:** ^1^ Health Sciences Center, Graduate Program in Public Health (PPGSC/UFES) at the Federal University of Espirito Santo, Vitória, Espírito Santo, Brazil; ^2^ Health Sciences Center, Graduate Program in Nutrition and Health (PPGNS/UFES) at the Federal University of Espirito Santo, Vitória, Espírito Santo, Brazil

**Keywords:** breast cancer, cancer symptom clusters, symptom management

## Abstract

**Background:**

Breast cancer is the most commonly diagnosed malignancy among women worldwide and a leading cause of cancer‐related morbidity. As treatment advances have improved survival rates, symptom management has become a key component of comprehensive cancer care. Cancer‐related symptoms often present in clusters rather than in isolation, potentially amplifying patient discomfort and negatively impacting quality of life. Identifying stage‐specific symptom cluster patterns may provide critical insights for developing personalized supportive care strategies. This study aimed to identify and compare the prevalence, intensity, and discomfort of symptom clusters in women with Stage I and Stage III nonmetastatic breast cancer.

**Method:**

This cross‐sectional study included 87 women aged > 18 years with histopathological diagnoses of Stages I–III breast cancer, undergoing any phase of antineoplastic treatment at an oncology hospital in Brazil. Symptoms were assessed using the Memorial Symptom Assessment Scale (MSAS). The bootstrap resampling method was used to estimate 95% confidence intervals (CIs) for prevalence ratios (PRs) of MSAS symptoms, stratified by cancer stage. Symptom clusters were identified using hierarchical and k‐means clustering analyses.

**Results:**

Among Stage I patients, the most prevalent symptoms were pain (68.6%), worrying (62.8%), difficulty sleeping (62.8%), and fatigue (60.8%). In Stage III patients, the most frequent symptoms were pain (72.0%), fatigue (66.7%), worrying (63.9%), and dry mouth (50.0%). Stage I patients had a higher prevalence of difficulty concentrating (PR = 1.50; *p* = 0.015), shortness of breath (PR = 1.51; *p* < 0.001), feeling sad (PR = 1.41; *p* = 0.002), and hair loss (PR = 1.60; *p* = 0.037) compared to those with Stage III disease. Four clusters were identified for Stage I patients—neuropsychological, gastrointestinal, neurocognitive, and psychological—and for Stage III patients—psychoneurocognitive, gastrointestinal, chemotherapy‐related, and neurocognitive.

**Conclusion:**

These findings highlight the heterogeneity of symptom experiences in women with nonmetastatic breast cancer, with distinct cluster profiles emerging at different disease stages. Understanding stage‐specific symptom patterns may inform more personalized and targeted supportive care strategies to improve quality of life and clinical outcomes in this population.

**Highlights**
• Among women with Stage I breast cancer, four distinct symptom clusters were identified: neuropsychological, gastrointestinal, neurocognitive, and psychological.
• In contrast, patients with Stage III breast cancer exhibited four different clusters: psychoneurocognitive, gastrointestinal, chemotherapy‐related, and neurocognitive.
• Notably, the symptom “difficulty concentrating” was significantly more prevalent in Stage I patients, occurring 1.50 times more frequently than in those with Stage III disease (PR = 1.50; *p* = 0.015).

## 1. Introduction

Cancer symptoms are dynamic and evolve throughout the course of breast cancer treatment, often exerting a profound impact on patients’ quality of life and functional capacity [1, 2]. These symptoms can interfere with treatment adherence and clinical outcomes, particularly when not adequately recognized or managed [3, 4]. Timely symptom assessment and effective management strategies have been shown to reduce hospitalization rates, lower healthcare costs, and alleviate physical and emotional distress [5].

Importantly, symptoms in oncology rarely occur in isolation. When multiple symptoms co‐occur—forming so‐called symptom clusters—their cumulative burden can have a more detrimental impact on patients’ well‐being than individual symptoms alone [6, 7]. Understanding how these symptoms coalesce can reveal shared physiological mechanisms and guide more targeted interventions, ultimately improving treatment outcomes and survivorship care [8–10].

Previous research has identified various symptom groupings in cancer patients, including associations between fatigue and sleep disturbances [11, 12], depression [12–14], anxiety [3, 15], pain [3, 16, 17], and gastrointestinal manifestations such as nausea and vomiting [18, 19]. For instance, anxiety often coexists with pain [3], nausea [18], and difficulty concentrating [20], underscoring the complexity of symptom experiences in breast cancer.

Symptom presentation and trajectory in breast cancer patients are influenced by factors such as menopausal status, comorbidities, timing of diagnosis, and the type and duration of antineoplastic therapy [3, 21]. During adjuvant treatment, particularly with agents like aromatase inhibitors, symptoms may intensify or shift over time, including exacerbation of menopausal symptoms, musculoskeletal pain, and cognitive decline [22–24].

With advances in diagnosis and therapy, an increasing proportion of breast cancer patients are surviving longer, shifting clinical focus toward survivorship and quality of life [25]. As such, there is growing recognition of the importance of understanding the symptom burden in this population and supporting long‐term well‐being [26, 27]. This necessitates a proactive, patient‐centered approach to managing the constellation of symptoms that affect both adherence and health‐related quality of life [26, 27].

Although symptom clusters have been studied in patients undergoing chemotherapy or radiotherapy, especially in advanced‐stage disease [28, 29], limited research has focused on primary symptom clustering in women with early‐ and locally advanced, nonmetastatic breast cancer. In particular, a few studies have compared how symptom clusters manifest in Stage I versus Stage III breast cancer. Addressing this gap, the present study aimed to identify and compare the prevalence, intensity, and discomfort of symptom clusters in women diagnosed with Stage I and Stage III nonmetastatic breast cancer.

## 2. Materials and Methods

### 2.1. Study Design and Setting

This cross‐sectional observational study was conducted in Inpatient Unit A of Afecc–Hospital Santa Rita de Cássia (HSRC), located in the southeastern region of Brazil. HSRC is a philanthropic institution founded in 1970 and is the only officially designated CACON (High‐Complexity Oncology Center) in the state of Espírito Santo. The hospital is nationally recognized as a reference center for cancer care and also provides general medical services to the broader population. Through a partnership with the Brazilian Unified Health System (Sistema Único de Saúde—SUS), more than 60% of its services are allocated to public healthcare delivery.

### 2.2. Ethical Considerations

This study was approved by the Research Ethics Committee of the Health Sciences Center of the Federal University of Espírito Santo on May 10, 2022 (Approval No. 5.400.652), in accordance with Resolution 466/2012 of the Brazilian National Health Council and the Declaration of Helsinki. All participants who met the eligibility criteria and agreed to participate provided written informed consent. Confidentiality, data anonymization, and the right to compensation for potential research‐related harms were guaranteed.

### 2.3. Eligibility and Sample Size

Inclusion criteria encompassed: (a) female patients aged ≥ 18 years; (b) histopathological diagnosis of Stages I, II, or III breast cancer (ICD‐10 code: C50); and (c) undergoing any phase of antineoplastic treatment. Exclusion criteria included the following: (a) Stage IV breast cancer; (b) patients in exclusive palliative care; and (c) hospitalizations for reconstructive surgery or clinical complications unrelated to breast cancer.

Although our original study protocol planned to include women diagnosed with Stages I, II, and III nonmetastatic breast cancer, no eligible patients with Stage II disease were hospitalized in the unit during the data collection period. As a result, no Stage II patients were available for recruitment. To avoid introducing bias arising from extremely small and unbalanced subgroups, the analysis was restricted to Stages I and III, which had sufficient representation for meaningful comparisons.

Sample size was estimated using the most recent incidence projections of female breast cancer by the Brazilian National Cancer Institute (INCA) for 2023–2025 [30], which estimated 74,000 new cases nationwide and an incidence rate of 84.46 per 100,000 women in the Southeast region. HSRC’s Inpatient Unit A recorded 225 breast cancer cases in 2019 and 182 in 2020, averaging 204 cases per year. To reduce the impact of COVID‐19‐related biases on hospital admissions, we based our calculation on the 2019 data only.

Using the formula for finite population sampling [31], we set *α* = 0.05, a confidence level of 95%, power of 80%, and a minimum proportion of 12%. This yielded a minimum required sample size of 85 patients. Ultimately, 87 eligible patients were recruited.

### 2.4. Data Collection Procedures and Measures

A structured sociodemographic and clinical questionnaire was developed by the principal investigators and organized into three sections: (I) Medical History, including oncological background, comorbidities, cardiovascular risk factors, lifestyle, diet, and physical activity; (II) General and Specific Physical Examination; and (III) Laboratory Data, such as complete blood count and capillary glucose, retrieved from medical records.

#### 2.4.1. Memorial Symptom Assessment Scale (MSAS)

To evaluate cancer symptoms, we employed the MSAS [32]. Designed by Portenoy and colleagues (1994) [32], the MSAS is a tool for identifying and tracking various symptoms in cancer patients. The scale quantifies symptoms by frequency, intensity, and discomfort, combining 32 symptoms. Patients assign numerical values ranging from 1 to 4 for symptom frequency and intensity over the past week, and 0 to 4 for discomfort levels. MSAS is divided into subscales: Psychological Symptoms (PSYCH) with 6 items and Physical Symptoms (PHYS H and PHYS L) with 26 items.

In the original validation, Cronbach’s alpha coefficients were 0.835 for psychological, 0.882 for high‐frequency physical, and 0.580 for low‐frequency physical symptoms [32]. The Brazilian Portuguese version demonstrated strong reliability, with Kappa coefficients ranging from 0.69 to 0.96 per item and subscale reliabilities of 0.84 (high‐frequency physical), 0.81 (low‐frequency physical), and 0.81 (psychological symptoms), with an overall distress index reliability of 0.78 [33]. Data collection took place over a 10‐month period, from June 2022 to March 2023.

### 2.5. Data Analysis

All statistical analyses were performed using R (v4.2.2) and RStudio (v2023.03.1). Categorical variables were reported as absolute and relative frequencies, and continuous variables were described using means, standard deviations, and medians. Pearson’s chi‐squared or Fisher’s exact tests [34] were used to assess associations between categorical variables, and Student’s *t*‐test was used for continuous variables. The bootstrap resampling method [35] was applied to estimate 95% confidence intervals (CIs) for prevalence ratios (PRs) of MSAS symptoms stratified by cancer stage. Symptom cluster analysis was conducted using both hierarchical clustering [36] and k‐means clustering techniques [37]. A *p*‐value of < 0.05 was considered statistically significant.

## 3. Results

### 3.1. Demographic and Clinical Characteristics of the Sample

Table 1 presents the demographic and clinical characteristics of the sample stratified by cancer stage. The study included 87 women diagnosed with nonmetastatic breast cancer, of whom 51 (59%) were classified as Stage I and 36 (41%) as Stage III. The mean age of participants was 60.0 years (SD = 10.16).

**TABLE 1:** Sociodemographic and clinical characteristics of women with nonmetastatic breast cancer by Stages I and III.

Variables	Stage I	Stage III	Total	*p*‐Value
	(*n* = 51)	(*n* = 36)	(*n* = 87)	
Age (years)				0.534^∗^
Mean (standard deviation)	60.61 (9.62)	59.22 (10.96)	60.03 (10.16)	
Median	61.00	60.00	60.00	
Age range				0.376^∗∗^
< 50 years	5 (9.80)	6 (16.67)	11 (12.64)	
50–64 years	30 (58.82)	16 (44.44)	46 (52.87)	
≥ 65 years old	16 (31.37)	14 (38.89)	30 (34.48)	
Self‐reported color				1000^∗∗^
White	19 (37.25)	14 (38.89)	33 (37.93)	
Non‐white	32 (62.75)	22 (61.11)	54 (62.07)	
Education				0.366^∗∗∗^
Illiterate	16 (31.37)	9 (25.00)	25 (28.74)	
Complete elementary school	22 (43.14	18 (50.00)	40 (45.98)	
Complete high school	4 (7.84)	6 (16.67)	10 (11.49)	
Higher education	9 (17.65)	3 (8.33)	12 (13.79)	
Marital status				0.624^∗∗^
Single	13 (25.49)	6 (16.67)	19 (21.84)	
Married	22 (43.14)	20 (55.56)	42 (48.28)	
Widow	8 (15.69)	6 (16.67)	14 (16.09)	
Divorced	8 (15.69)	4 (11,11)	12 (13.79)	
Children				0.107^∗∗^
None	7 (13.73)	8 (22.22)	15 (17.24)	
1	17 (33.33)	5 (13.89)	22 (25.29)	
≥ 2	27 (52.94)	23 (63.89)	50 (57.47)	
Smoking				0.461^∗∗∗^
No	45 (88.24)	34 (94.44)	79 (90.80)	
Yes	6 (11.76)	2 (5.56)	8 (9.20)	
Alcohol consumption				0.058^∗∗^
No	47 (92.16)	27 (75.00)	74 (86.05)	
Yes	4 (7.84)	9 (25.00)	13 (13.95)	
Histological type				0.900^∗∗^
Invasive carcinoma	17 (33.33)	13 (36.11)	30 (34.48)	
Ductal in situ	19 (37.25)	14 (38.89)	33 (37.93)	
Lobular in situ	15 (29.41)	9 (25.00)	24 (27.59)	
Current cancer diagnosis				0.409^∗∗∗^
Breast malignant neoplasm	16 (31.37)	17 (47.22)	33 (37.93)	
Central breast malignant neoplasm	13 (25.49)	5 (13.89)	18 (20.69)	
Malignant neoplasm of the upper inner quadrant of the breast	10 (19.61)	4 (11,11)	14 (16.09)	
Malignant neoplasm of the lower inner quadrant of the breast	1 (1.96)	1 (2.78)	2 (2.30)	
Malignant neoplasm of the upper outer quadrant of the breast	1 (1.96)	1 (2.78)	2 (2.30)	
Malignant neoplasm of the lower outer quadrant of the breast	5 (9.80)	1 (2.78)	6 (6.90)	
Malignant neoplasm of the axillary portion of the breast	3 (5.88)	4 (11,11)	7 (8.07)	
Malignant breast neoplasm with invasive lesion	2 (3.92)	3 (8.33)	5 (5.75)	

^∗^Student’s *t*‐test.

^∗∗^Pearson chi‐square test.

^∗∗∗^Fisher’s exact test.

Most participants self‐identified as non‐white (Black or mixed race), had completed primary education (*n* = 40; 45.98%), were married (*n* = 42; 48.28%), had two or more children (*n* = 50; 57.47%), and reported no history of smoking or alcohol consumption. The most common histological subtype was invasive ductal carcinoma (*n* = 33; 37.93%), followed by invasive carcinoma not otherwise specified (*n* = 30; 34.48%).

No statistically significant differences were observed between patients with Stage I and Stage III breast cancer in terms of sociodemographic or clinical variables (*p* > 0.05 for all comparisons).

Regarding antineoplastic treatment, women with Stage III breast cancer predominantly received multimodal therapy, including anthracycline‐ and taxane‐based chemotherapy, adjuvant radiotherapy, and endocrine therapy when indicated. A subset of HER2‐positive patients also received targeted anti‐HER2 therapy (trastuzumab). In contrast, most patients with Stage I disease had undergone breast‐conserving or radical surgery followed by endocrine therapy, with fewer requiring combined chemotherapy regimens or radiotherapy. As expected, treatment intensity and cumulative toxicity were higher among Stage III patients. This therapeutic profile contextualizes the distinct symptom patterns identified between the groups and supports the biological plausibility of the observed clusters.

### 3.2. Prevalence of Cancer Symptom Clusters

Table 2 presents an overview of symptom prevalence among women diagnosed with Stage I and Stage III nonmetastatic breast cancer. In the Stage I group, the most commonly reported symptoms were pain (68.63%), worrying (62.75%), sleep disturbances (62.75%), and fatigue (60.78%). Among women with Stage III disease, the most prevalent symptoms included pain (72.00%), fatigue (66.67%), worrying (63.89%), and dry mouth (50.00%).

**TABLE 2:** Prevalence of cancer symptoms in women with breast cancer by Stages I and III.

MSAS symptoms		Stage I		Stage III		PR	Estimated 95% CI	*p*‐Value
		Prevalence		Prevalence				
		*n*	%	*n*	%			
S1	Difficulty concentrating	17	33.33	8	22.22	1.50	1.52–1.73	**0.015**
S2	Pain	35	68.63	26	72.22	0.95	0.93–0.98	0.680
S3	Fatigue	31	60.78	24	66.67	0.91	0.90–0.95	0.312
S4	Cough	12	23.53	14	38.89	0.61	0.59–0.65	0.369
S5	Feeling nervous	25	49.02	16	44.44	1.10	1.10–1.18	0.096
S6	Dry mouth	27	52.94	18	50.00	1.06	1.07–1.14	**0.011**
S7	Nausea	17	33.33	15	41.67	0.80	0.79–0.86	0.149
S8	Feeling drowsy	19	37.25	13	36.11	1.03	1.05–1.19	**0.015**
S9	Numbness/tingling in hands	18	35.29	16	44.44	0.79	0.77–0.83	0.664
S10	Difficulty sleeping	32	62.75	17	47.22	1.33	1.33–1.41	0.051
S11	Feeling bloated	18	35.29	16	44.44	0.79	0.81–0.87	**0.007**
S12	Problems with urination	0	0.00	0	0.00	—	—	—
S13	Vomiting	9	17.65	6	16.67	1.06	1.12–1.43	**0.006**
S14	Shortness of breath	15	29.41	7	19.44	1.51	1.69–2.02	**<** **0.001**
S15	Diarrhea	15	29.41	16	44.44	0.66	0.66–0.71	0.065
S16	Feeling sad	22	43.14	11	30.56	1.41	1.45–1.59	**0.002**
S17	Sweats	25	49.02	10	27.78	1.76	1.88–2.09	**<** **0.001**
S18	Worrying	32	62.75	23	63.89	0.98	0.98–1.03	**0.037**
S19	Problems with sexual interest or activity	9	17.65	6	16.67	1.06	1.11–1.31	**0.004**
S20	Itching	7	13.73	8	22.22	0.62	0.64–0.75	**0.004**
S21	Lack of appetite	20	39.22	16	44.44	0.88	0.88–0.96	**0.048**
S22	Dizziness	17	33.33	11	30.56	1.09	1.12–1.26	**0.007**
S23	Difficulty swallowing	15	29.41	8	22.22	1.32	1.40–1.66	**0.002**
S24	Feeling irritable	19	37.25	11	30.56	1.22	1.29–1.43	**<** **0.001**
S25	Mouth sores	10	19.61	3	8.33	2.35	2.72–3.33	**<** **0.001**
S26	Change in the way food tastes	19	37.25	13	36.11	1.03	1.04–1.13	**0.022**
S27	Weight loss	24	47.06	12	33.33	1.41	1.47–1.60	**<** **0.001**
S28	Hair loss	25	49.02	11	30.56	1.60	1.61–1.75	**0.037**
S29	Constipation	25	49.02	13	36.11	1.36	1.37–1.48	**0.017**
S30	Swelling of arms or legs	10	19.61	7	19.44	1.01	1.15–1.34	**<** **0.001**
S31	“I don’t look like myself”	15	29.41	11	30.56	0.96	0.99–1.09	**0.002**
S32	Changes in skin	9	17.65	3	8.33	2.12	2.51–3.08	**<** **0.001**

*Note:* The values in bold in the table represent statistically significant results.

Abbreviations: CI = confidence interval, MSAS = Memorial Symptom Assessment Scale, PR = prevalence ratio.

Notably, the prevalence of “difficulty concentrating” was 1.50 times higher in Stage I patients compared to those with Stage III breast cancer (PR = 1.50; *p* = 0.015). Similarly, Stage I patients exhibited significantly higher PRs for shortness of breath (PR = 1.51; *p* < 0.001), feeling sad (PR = 1.41; *p* = 0.002), and hair loss (PR = 1.60; *p* = 0.037).

Additionally, the prevalence of mouth sores and constipation was significantly greater in Stage I patients, with PRs of 2.35 (*p* < 0.001) and 1.36 (*p* = 0.017), respectively. In contrast, Stage III patients had a lower prevalence of bloating (PR = 0.79; *p* = 0.007) and concerns/worrying (PR = 0.98; *p* = 0.037) when compared to their Stage I counterparts.

Within this sample, several primary symptom clusters emerged, characterized by their frequency, intensity, and discomfort across different stages of breast cancer. Among women with Stage I disease, four distinct symptom clusters were identified:

⁃ Neuropsychological cluster: fatigue, drowsiness, difficulty sleeping, nervousness, and the sensation of not looking like oneself.
⁃ Gastrointestinal cluster: nausea, vomiting, bloating, difficulty swallowing, loss of appetite, and changes in taste.
⁃ Neurocognitive cluster: pain, dry mouth, dizziness, and numbness/tingling in the hands.
⁃ Psychological cluster: sadness, worrying, and nervousness.

The detailed symptom cluster profiles identified through the K‐means clustering method for hospitalized Stage I breast cancer patients are illustrated in​ Table 3.

**TABLE 3:** Clustering of cancer symptoms in women with Stage I breast cancer according to frequency, intensity, and discomfort.

Clusters	Symptoms of women with Stage I breast cancer		
	Frequency	Intensity	Discomfort
1	(S2) Pain	(S3) Fatigue	(S4) Cough
	(S6) Dry mouth	(S9) Numbness/tingling in hands	(S7) Nausea
	(S15) Diarrhea	(S10) Difficulty sleeping	(S9) Numbness/tingling in hands
	(S16) Feeling sad	(S16) Feeling sad	(S11) Feeling bloated
	(S17) Sweats	(S18) Worrying	(S20) Itching
	(S18) Worrying	(S27) Weight loss	(S23) Difficulty swallowing
	(S22) Dizziness	(S28) Hair loss	(S25) Mouth sores
	(S26) Change in the way food tastes	(S31) “I don’t look like myself”	(S26) Change in the way food tastes
	(S27) Weight loss		(S31) “I don’t look like myself”
	(S28) Hair loss		(S32) Changes in skin

2	(S1) Difficulty concentrating	(S1) Difficulty concentrating	(S2) Pain
	(S4) Cough	(S5) Feeling nervous	(S3) Fatigue
	(S7) Nausea	(S7) Nausea	(S5) Feeling nervous
	(S11) Feeling bloated	(S11) Feeling bloated	(S6) Dry mouth
	(S12) Problems with urination	(S13) Vomiting	(S10) Difficulty sleeping
	(S13) Vomiting	(S14) Shortness of breath	(S16) Feeling sad
	(S14) Shortness of breath	(S15) Diarrhea	(S18) Worrying
	(S19) Problems with sexual interest or activity	(S17) Sweats	
	(S20) Itching	(S19) Problems with sexual interest or activity	
	(S21) Lack of appetite	(S21) Lack of appetite	
	(S23) Difficulty swallowing	(S22) Dizziness	
	(S30) Swelling of arms or legs	(S23) Difficulty swallowing	
	(S32) Changes in skin	(S24) Feeling irritable	
		(S26) Change in the way food tastes	

3	(S3) Fatigue	(S4) Cough	(S13) Vomiting
	(S8) Feeling drowsy	(S12) Problems with urination	(S14) Shortness of breath
	(S10) Difficulty sleeping	(S20) Itching	(S15) Diarrhea
	(S24) Feeling irritable	(S30) Swelling of arms or legs	(S17) Sweats
	(S29) Constipation	(S32) Changes in skin	(S21) Lack of appetite
			(S22) Dizziness
			(S24) Feeling irritable
			(S29) Constipation

4	(S5) Feeling nervous	(S2) Pain	(S1) Difficulty concentrating
	(S9) Numbness/tingling in hands	(S6) Dry mouth	(S8) Feeling drowsy
	(S25) Mouth sores	(S8) Feeling drowsy	(S12) Problems with urination
	(S31) “I don’t look like myself”	(S25) Mouth sores	(S19) Problems with sexual interest or activity
		(S29) Constipation	(S27) Weight loss
			(S28) Hair loss
			(S30) Swelling of arms or legs

*Note:* S1= Symptom 1 of the Memorial Symptom Assessment Scale (MSAS).

In women with Stage III breast cancer, four distinct symptom clusters were also observed:

⁃ Psychoneurocognitive cluster: pain, fatigue, dry mouth, sadness, worrying, and the sensation of not looking like oneself.
⁃ Gastrointestinal cluster: diarrhea, mouth sores, constipation, and difficulty swallowing.
⁃ Chemotherapy‐related cluster: nausea, vomiting, and skin changes.
⁃ Neurocognitive cluster: difficulty sleeping, drowsiness, and dizziness.

A comprehensive depiction of these symptom clusters, as identified by K‐means clustering in hospitalized Stage III breast cancer patients, is provided in Table 4.

**TABLE 4:** Clustering of cancer symptoms in women with Stage III breast cancer according to frequency, intensity, and discomfort.

Clusters	Symptoms of women with Stage III breast cancer		
	Frequency	Intensity	Discomfort
1	(S5) Feeling nervous	(S5) Feeling nervous	(S3) Fatigue
	(S9) Numbness/tingling in hands	(S9) Numbness/tingling in hands	(S5) Feeling nervous
	(S15) Diarrhea	(S20) Itching	(S6) Dry mouth
	(S20) Itching	(S22) Dizziness	(S7) Nausea
	(S23) Difficulty swallowing	(S29) Constipation	(S9) Numbness/tingling in hands
	(S25) Mouth sores	(S31) “I don’t look like myself”	(S10) Difficulty sleeping
	(S29) Constipation		(S11) Feeling bloated
			(S16) Feeling sad
			(S28) Hair loss

2	(S2) Pain	(S1) Difficulty concentrating	(S13) Vomiting
	(S3) Fatigue	(S12) Problems with urination	(S14) Shortness of breath
	(S4) Tosse	(S13) Vomiting	(S15) Diarrhea
	(S6) Cough	(S14) Shortness of breath	(S17) Sweats
	(S16) Feeling sad	(S19) Problems with sexual interest or activity	(S23) Difficulty swallowing
	(S18) Worrying	(S23) Difficulty swallowing	(S24) Feeling irritable
	(S21) Lack of appetite	(S25) Mouth sores	
	(S26) Change in the way food tastes	(S30) Swelling of arms or legs	
	(S27) Weight loss	(S32) Changes in skin	
	(S28) Hair loss		
	(S31) “I don’t look like myself”		

3		(S2) Pain	(S1) Difficulty concentrating
	(S8) Feeling drowsy	(S7) Nausea	(S8) Feeling drowsy
	(S10) Difficulty sleeping	(S8) Feeling drowsy	(S19) Problems with sexual interest or activity
	(S11) Feeling bloated	(S10) Difficulty sleeping	(S20) Itching
		(S11) Feeling bloated	(S21) Lack of appetite
		(S17) Sweats	(S26) Change in the way food tastes
		(S21) Lack of appetite	(S27) Weight loss
		(S24) Feeling irritable	(S29) Constipation
			(S30) Swelling of arms or legs

4	(S1) Difficulty concentrating	(S3) Fatigue	(S2) Pain
	(S7) Nausea	(S4) Cough	(S4) Cough
	(S12) Problems with urination	(S6) Dry mouth	(S12) Problems with urination
	(S13) Vomiting	(S15) Diarrhea	(S18) Worrying
	(S14) Shortness of breath	(S16) Feeling sad	(S22) Dizziness
	(S17) Sweats	(S18) Worrying	(S25) Mouth sores
	(S19) Problems with sexual interest or activity	(S26) Change in the way food tastes	(S31) “I don’t look like myself”
	Dizziness	(S27) Weight loss	(S32) Changes in skin
	Feeling irritable	(S28) Hair loss	
	Swelling of arms or legs		
	Changes in skin		

*Note:* S1 = Symptom 1 of the Memorial Symptom Assessment Scale (MSAS).

Figures 1(a), 2(a) present the dendrograms generated using hierarchical clustering methods, reflecting the structure of symptom relationships in patients with Stage I and Stage III breast cancer, respectively.

FIGURE 1MSAS symptom clusters by frequency, intensity, and discomfort among hospitalized women with Stage I breast cancer.

**Figure:**
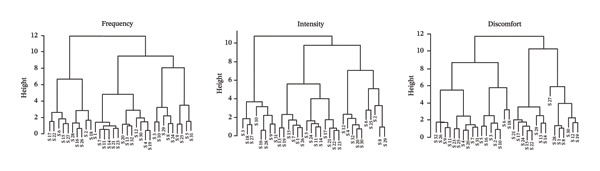
(a)

**Figure:**
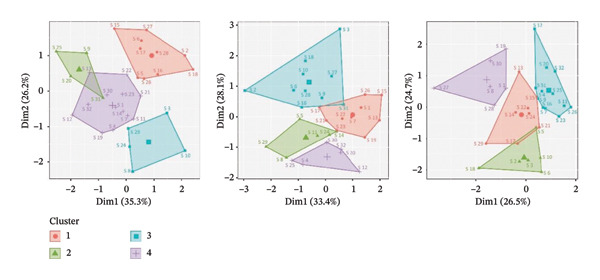
(b)

FIGURE 2MSAS symptom clusters by frequency, intensity, and discomfort among hospitalized women with Stage III breast cancer.

**Figure:**
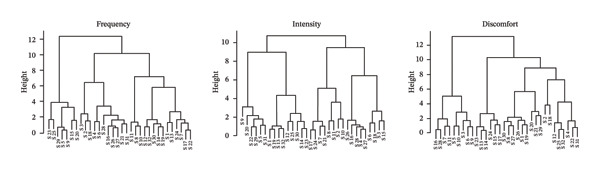
(a)

**Figure:**
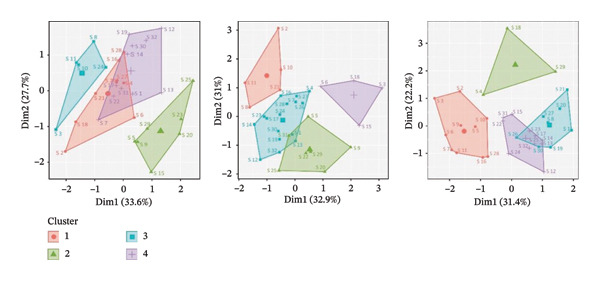
(b)

Figures 1(b), 2(b) display the four primary symptom clusters derived from K‐means clustering, corresponding to breast cancer Stages I and III.

## 4. Discussion

This study provides novel insights and a comparative analysis of the prevalence, intensity, and discomfort of cancer symptom clusters among women with nonmetastatic Stage I and Stage III breast cancer. In Stage I patients, the most prevalent symptoms were pain (68.63%), worrying (62.75%), sleep disturbances (62.75%), and fatigue (60.78%). In contrast, Stage III patients most frequently reported pain (72.00%), fatigue (66.67%), worrying (63.89%), and dry mouth (50.00%).

Distinct cluster configurations emerged according to disease stage. Among Stage I patients, four primary symptom clusters were identified: a neuropsychological cluster (fatigue, feeling drowsy, difficulty sleeping, feeling nervous, and “I don’t look like myself”), a gastrointestinal cluster (nausea, vomiting, bloating, difficulty swallowing, lack of appetite, and altered taste), a neurocognitive cluster (pain, dry mouth, dizziness, and numbness/tingling in hands), and a psychological cluster (feeling sad, worrying, and feeling nervous). In Stage III patients, symptom clusters were characterized as psychoneurocognitive, gastrointestinal, chemotherapy‐related, and neurocognitive.

These cluster patterns align with findings from other studies involving cancer patients receiving antineoplastic treatment. For example, a study of 399 gastrointestinal cancer patients undergoing chemotherapy identified four symptom clusters based on frequency, intensity, and severity: a neuropsychological cluster, chemotherapy‐related cluster, gastrointestinal cluster, and a weight change‐related cluster [38]. Fatigue emerged as the most prevalent and severe symptom, while the item “I don’t look like myself” was the most distressing—consistent with previous findings among breast cancer patients [39–41]. In our study, fatigue was similarly identified as the second most frequent symptom in both Stage I and Stage III groups.

The prevalence of cancer‐related fatigue (CRF) varies widely in the literature, ranging from 10% to 99% [42], with some studies reporting rates as high as 90.3% among patients receiving chemotherapy [43]. Fatigue is recognized as a debilitating and multifactorial symptom with a profound negative impact on quality of life [44]. Studies consistently report moderate to severe fatigue in 30%–60% of patients, with levels high enough to warrant interruption of cancer treatment in some cases [45].

In a study examining the impact of fatigue on quality of life in breast cancer patients undergoing chemotherapy, binary logistic regression analysis identified several significant predictors of fatigue severity. Key factors included cancer stage (*p* = 0.026), chemotherapy dose delay (*p* = 0.038), chemotherapy regimen (*p* = 0.003), chemotherapy dose reduction (*p* = 0.011), and ethnicity (*p* = 0.027) [46]. Notably, a negative association was observed between cancer stage—particularly Stage II—and fatigue severity, indicating that more advanced disease stages were linked to heightened fatigue levels (*p* < 0.005). Patients receiving combined chemotherapy regimens were found to be four times more likely to experience severe fatigue than those on monotherapy. Additionally, the absence of chemotherapy dose reduction was associated with a 22.3‐fold increased likelihood of experiencing high fatigue levels [46].

Another study investigating the longitudinal dynamics of fatigue and anxiety demonstrated that breast cancer patients continued to experience clinically significant levels of both symptoms up to 6 months postchemotherapy, with a substantial negative impact on their overall quality of life [47].

In a study designed to evaluate the prevalence of cancer‐related symptoms and the formation of symptom clusters at different phases of breast cancer, researchers stratified participants into three distinct clinical stages: Phase I—patients with early‐stage disease postsurgery but prior to systemic therapy; Phase II—patients with Stage I–III disease who had completed surgery and adjuvant treatment; and Phase III—patients with Stage IV metastatic breast cancer [3]. Across these phases, three major symptom clusters were identified, and notably, fatigue persisted as a central symptom in all groups [3]. Interestingly, even in Phase I—before the initiation of adjuvant therapy—patients reported pain as a prevalent symptom. This finding is particularly relevant to the present study, which also identified pain and fatigue as dominant symptoms in early‐stage (Stage I) patients.

These findings reinforce the complexity and persistence of fatigue and other co‐occurring symptoms throughout the breast cancer care continuum. The presence of symptom clusters—even before the onset of systemic therapy—highlights the importance of early symptom assessment and management strategies tailored to specific disease stages.

Previous studies have consistently documented the co‐occurrence of sleep disturbances and cancer‐related fatigue in oncology patients [6, 48, 49]. Notably, proinflammatory cytokines—particularly IL‐1β, IL‐6, and TNF‐α—have been implicated in the pathophysiology of fatigue and alterations in sleep patterns [50, 51]. In a study involving 28 women with breast cancer at various stages, higher levels of fatigue were positively correlated with concentrations of the IL‐1 receptor antagonist (IL‐1ra; *p* < 0.03) [52]. Furthermore, a prospective longitudinal study conducted among early‐stage breast cancer patients (*n* = 52) undergoing adjuvant radiotherapy demonstrated a significant association between fatigue and elevated IL‐6 levels during the fifth week of treatment (*p* = 0.03) [53]. Genetic polymorphisms related to cytokine regulation have also been linked to fatigue. For example, the presence of at least one IL1β‐511 allele (95% CI: 0.91–16.6; *p* = 0.007) and homozygosity for the IL6‐174 genotype variant (G/G or C/C; 95% CI: 1.12–17.9; *p* = 0.027) were identified as predictors of increased fatigue severity in breast cancer patients [54].

In our study, the neuropsychological symptom cluster emerged as the most prominent among Stage I breast cancer patients. This finding aligns with previous research demonstrating the prevalence of neuropsychological clusters among breast cancer patients, characterized by symptoms such as worry, sadness, nervousness, irritability, difficulty concentrating, fatigue, and drowsiness [39, 41, 51, 55, 56]. The biological plausibility of these clusters is supported by studies linking neuroimmune processes and inflammatory cytokine release—such as IL‐1β, IL‐6, IL‐8, IL‐10, IL‐12p70, TNF‐α, and IFN‐γ—to neuropsychological symptoms, including depressed mood, heightened pain sensitivity, sleep disturbances, and fatigue [9, 10, 50, 57–60]. Within the tumor microenvironment, cytokine signaling plays a key role in regulating the host’s response to stress and damage, and enhanced understanding of these mechanisms could provide critical insight into symptomatology and prognostic trajectories in oncology [61–64]. Additionally, cognitive complaints frequently coincide with mood disturbances, depression, and anxiety—further reinforcing the conceptual framework of the neuropsychological cluster [65].

A chemotherapy‐related symptom cluster was notably present among patients with Stage III disease in our cohort. This cluster was defined by nausea, vomiting, and dermatologic changes, aligning with findings from previous studies exploring chemotherapy‐induced toxicities [38, 55, 66]. One investigation focused on early‐stage lung cancer patients revealed that both the number of comorbidities and exposure to chemotherapy were predictive of a higher prevalence and intensity of these symptoms [67].

The gastrointestinal symptom cluster—characterized by nausea, lack of appetite, and taste alterations—was identified in both Stage I and Stage III breast cancer patients. This observation echoes findings from prior research, in which gastrointestinal symptom clusters were commonly reported among breast cancer patients across treatment stages [38, 39, 41, 51, 68]. A consistent convergence of symptoms—nausea, vomiting, anorexia, and diarrhea—has also been reported in eight separate studies analyzing symptom clusters in patients who had completed treatment [21, 39, 69–74]. Furthermore, a recent update of a systematic review of symptom clusters in breast cancer patients during and after treatment highlighted the gastrointestinal, psychological, pain–fatigue–sleep disturbance, and menopausal clusters as the most frequently reported [75]. These findings are partially corroborated by the current study, further supporting the validity of our cluster categorizations.

## 5. Clinical Implications

The identification of distinct symptom clusters in women with Stage I and Stage III nonmetastatic breast cancer reinforces the importance of adopting stage‐specific strategies for symptom assessment and management in clinical oncology. Tailored supportive care interventions based on cluster profiles may enhance symptom control, improve quality of life, and promote better adherence to treatment protocols [76–80].

Furthermore, these findings contribute to the advancement of precision medicine/nursing by emphasizing the potential integration of symptom clusters with biological markers [81–83]. Future clinical protocols may benefit from the incorporation of biomarker analyses—such as inflammatory cytokines and genomic profiles—to predict symptom burden, stratify risk, and personalize supportive care. Implementing routine monitoring of symptom clusters, in conjunction with biomarker data, may support the development of dynamic, patient‐centered care models that anticipate and mitigate adverse effects throughout the cancer treatment continuum.

### 5.1. Limitations and Strengths

This study has some limitations that should be considered. First, it was conducted in a single oncology reference center in Brazil, which may limit the external validity and generalizability of the findings to other populations or healthcare systems. Second, the cross‐sectional design captured patients at different phases of their treatment trajectories and under various antineoplastic regimens, potentially influencing the symptom burden and cluster composition. Longitudinal studies are warranted to examine the temporal stability and evolution of symptom clusters over time. Third, the sample size—particularly among women with Stage III breast cancer—was relatively modest, which may have limited the statistical power for detecting more subtle differences. Additionally, no patients with Stage II disease were hospitalized or eligible during the recruitment window, which prevented their inclusion. Although this did not compromise the study objectives, it limited the comparison to Stages I and III.

Despite these limitations, the study also presents notable strengths. It is one of the few investigations to compare symptom cluster profiles specifically between Stage I and Stage III nonmetastatic breast cancer patients in a middle‐income country context. The use of a validated and culturally adapted instrument (the MSAS) for multidimensional symptom assessment enhances the reliability of the findings. Additionally, the application of both hierarchical and k‐means clustering methods, along with bootstrap techniques, provided methodological robustness and strengthened the identification and interpretation of symptom patterns. These findings contribute valuable insights into the growing body of literature on symptom clustering in oncology and offer implications for more personalized, stage‐specific supportive care interventions.

## 6. Conclusion

This study demonstrates that women with nonmetastatic breast cancer present distinct symptom cluster profiles according to disease stage. Stage I patients exhibited four clusters—neuropsychological, gastrointestinal, neurocognitive, and psychological—while Stage III patients revealed psychoneurocognitive, gastrointestinal, chemotherapy‐related, and neurocognitive clusters. Each cluster was characterized by specific symptom patterns, intensities, and levels of discomfort.

These findings underscore the clinical relevance of identifying symptom clusters tailored to cancer stage, contributing to more nuanced and effective symptom assessment and management strategies. Future longitudinal studies are warranted to examine the temporal stability of these clusters, investigate their physiological underpinnings, and determine their impact on treatment adherence, functional outcomes, nutritional status, and health‐related quality of life.

Ultimately, our results advocate for the integration of symptom cluster analysis into routine clinical care and the development of stage‐specific, patient‐centered interventions. Moving beyond descriptive analyses, the next step involves evaluating targeted interventions aimed at mitigating the burden of co‐occurring symptoms—an essential advancement for multidisciplinary oncology care.

## Author Contributions

Luís Carlos Lopes‐Júnior and Wesley Rocha Grippa designed the research; Roberto Júnio Gomes Silva, Luís Carlos Lopes‐Júnior, Luiz Cláudio Barreto Silva Neto, Naira Santos D’Agostini, Livia Machado Giacomin, and Raphael Manhães Pessanha conducted the research; Wesley Rocha Grippa and Luís Carlos Lopes‐Júnior analyzed the data; Roberto Júnio Gomes Silva, Etreo Junior Carneiro da Silva Minarini, Raphael Manhães Pessanha, Jonathan Grassi, Leticia Batista de Azevedo, Karla Anacleto Vasconcellos, Livia Machado Giacomin, Oscar Geovanny Enriquez‐Martinez, and Luiz Cláudio Barreto Silva wrote this article. Luís Carlos Lopes‐Júnior had primary responsibility for final content. Luís Carlos Lopes‐Júnior and Etreo Junior Carneiro da Silva Minarini contributed equally to this manuscript and share first authorship.

## Funding

This study was supported by Fundação de Amparo à Pesquisa e Inovação do Espírito Santo (FAPES), Call FAPES No. 03/2021 – Universal. FAPES Process No.: 432/2021; and the Conselho Nacional de Desenvolvimento Científico e Tecnológico (CNPq), Research Productivity Fellowship (PQ2), Process No.: 311427/2023‐5. Dr. Luís Carlos Lopes‐Júnior.

## Disclosure

All authors read and approved the final manuscript.

## Conflicts of Interest

The authors declare no conflicts of interest.

## Data Availability

The data that support the findings of this study are available upon request from the corresponding author. The data are not publicly available due to privacy or ethical restrictions.

## References

[1] Aktas A. , Walsh D. , and Hu B. , Cancer Symptom Clusters: An Exploratory Analysis of Eight Statistical Techniques, Journal of Pain and Symptom Management. (December 2014) 48, no. 6, 1254–1266, 10.1016/j.jpainsymman.2014.02.006, 2-s2.0-84919844730. 24747226

[2] Whisenant M. , Wong B. , Mitchell S. A. , Beck S. L. , and Mooney K. , Symptom Trajectories Are Associated With Co-Occurring Symptoms During Chemotherapy for Breast Cancer, Journal of Pain and Symptom Management. (February 2019) 57, no. 2, 183–189, 10.1016/j.jpainsymman.2018.11.010, 2-s2.0-85058414091. 30453052 PMC6348053

[3] Bender C. M. , Ergÿn F. S. , Rosenzweig M. Q. , Cohen S. M. , and Sereika S. M. , Symptom Clusters in Breast Cancer Across 3 Phases of the Disease, Cancer Nursing. (May 2005) 28, no. 3, 219–225, 10.1097/00002820-200505000-00011. 15915067

[4] Erdemsoy Karahan B. and Izgu N. , Impact of Symptom Burden and Self-Efficacy on Functional Status in Advanced Breast Cancer Patients: A Path Analysis, Nursing and Health Sciences. (July 2023) 11, no. 3, 354–364, 10.1111/nhs.13033. 37431570

[5] Cleeland C. S. , Mendoza T. R. , Wang X. S. et al., Assessing Symptom Distress in Cancer Patients: The M.D. Anderson Symptom Inventory, Cancer. (October 2000) 89, no. 7, 1634–1646, 10.1002/1097-0142(20001001)89. 11013380

[6] Dodd M. , Janson S. , Facione N. et al., Advancing the Science of Symptom Management, Journal of Advanced Nursing. (March 2001) 33, no. 5, 668–676, 10.1046/j.1365-2648.2001.01697.x, 2-s2.0-18044404277. 11298204

[7] Velasco Y. R. J. , Carvalho Fernandes A. F. , Miranda M. S. et al., Palliative Care in the Treatment of Women With Breast Cancer: A Scoping Review Protocol, BMJ Open. (June 2023) 13, no. 6, 10.1136/bmjopen-2022-068236. PMC10410954 37380202

[8] Cleeland C. S. , Bennett G. J. , Dantzer R. et al., Are the Symptoms of Cancer and Cancer Treatment due to a Shared Biologic Mechanism? A Cytokine-Immunologic Model of Cancer Symptoms, Cancer. (June 2003) 97, no. 11, 2919–2925, 10.1002/cncr.11382, 2-s2.0-0038182619. 12767108

[9] Kim H. J. , Barsevick A. M. , Fang C. Y. , and Miaskowski C. , Common Biological Pathways Underlying the Psychoneurological Symptom Cluster in Cancer Patients, Cancer Nursing. (November 2012) 35, no. 6, E1–E20, 10.1097/NCC.0b013e318233a811, 2-s2.0-84868304280. 22228391

[10] Lopes-Júnior L. C. , Ferrarini T. , Pires L. B. C. , Rodrigues J. G. , Salaroli L. B. , and Nunes K. Z. , Cancer Symptom Clusters in Adult Patients Undergoing Chemotherapy: A Systematic Review and Meta-Analysis Protocol, PLoS One. (September 2022) 17, no. 9, 10.1371/journal.pone.0273411. PMC9447873 36067147

[11] Ancoli-Israel S. , Liu L. , Marler M. R. et al., Fatigue, Sleep, and Circadian Rhythms Prior to Chemotherapy for Breast Cancer, Supportive Care in Cancer. (March 2006) 14, no. 3, 201–209, 10.1007/s00520-005-0861-0, 2-s2.0-32644478255. 16010529 PMC1599708

[12] Liu L. , Fiorentino L. , Natarajan L. et al., Pre-Treatment Symptom Cluster in Breast Cancer Patients is Associated With Worse Sleep, Fatigue and Depression During Chemotherapy, Psycho-Oncology. (February 2009) 18, no. 2, 187–194, 10.1002/pon.1412, 2-s2.0-66149112567. 18677716 PMC2762479

[13] Berger A. M. , Wielgus K. , Hertzog M. , Fischer P. , and Farr L. , Patterns of Circadian Activity Rhythms and Their Relationships With Fatigue and Anxiety/Depression in Women Treated With Breast Cancer Adjuvant Chemotherapy, Supportive Care in Cancer. (January 2010) 18, no. 1, 105–114, 10.1007/s00520-009-0636-0, 2-s2.0-71349084794. 19381692

[14] Bower J. E. , Ganz P. A. , Irwin M. R. , Kwan L. , Breen E. C. , and Cole S. W. , Inflammation and Behavioral Symptoms After Breast Cancer Treatment: Do Fatigue, Depression, and Sleep Disturbance Share a Common Underlying Mechanism?, Journal of Clinical Oncology. (September 2011) 29, no. 26, 3517–3522, 10.1200/JCO.2011.36.1154, 2-s2.0-80053009661. 21825266 PMC3179252

[15] Dragomir B. I. and Fodoreanu L. , Correlations Between State Anxiety and Quality of Life in Metastatic Breast Cancer Patients, Revista Medico-Chirurgicala Societatil di Medicl sl Naturalisti din Iasi. (July 2013) 117, no. 3, 610–615. 24502024

[16] Nieboer P. , Buijs C. , Rodenhuis S. et al., Fatigue and Relating Factors in High-Risk Breast Cancer Patients Treated With Adjuvant Standard or High-Dose Chemotherapy: A Longitudinal Study, Journal of Clinical Oncology. (November 2005) 23, no. 33, 8296–8304, 10.1200/JCO.2005.10.167, 2-s2.0-33644667529. 16219926

[17] Bjerkeset E. , Röhrl K. , and Schou-Bredal I. , Symptom Cluster of Pain, Fatigue, and Psychological Distress in Breast Cancer Survivors: Prevalence and Characteristics, Breast Cancer Research and Treatment. (February 2020) 180, no. 1, 63–71, 10.1007/s10549-020-05522-8. 31938939 PMC7031174

[18] Molassiotis A. , Yam B. M. , Yung H. , Chan F. Y. , and Mok T. S. , Pretreatment Factors Predicting the Development of Postchemotherapy Nausea and Vomiting in Chinese Breast Cancer Patients, Supportive Care in Cancer. (March 2002) 10, no. 2, 139–145, 10.1007/s00520-001-0321-4, 2-s2.0-0036933172. 11862503

[19] Huang X. , Li X. , Li J. et al., Chemotherapy-Induced Nausea and Vomiting in Breast Cancer Patients: A Multicenter Prospective Observational Study, Asia-Pacific Journal of Oncology Nursing. (May 2021) 8, no. 4, 433–437, 10.4103/apjon.apjon-2120. 34159237 PMC8186383

[20] Merriman J. D. , Sereika S. M. , Brufsky A. M. et al., Trajectories of Self-Reported Cognitive Function in Postmenopausal Women During Adjuvant Systemic Therapy for Breast Cancer, Psycho-Oncology. (January 2017) 26, no. 1, 44–52, 10.1002/pon.4009, 2-s2.0-84950115422. 26486371 PMC4969219

[21] Li H. , Sereika S. M. , Marsland A. L. , Conley Y. P. , and Bender C. M. , Symptom Clusters in Women With Breast Cancer During the First 18 Months of Adjuvant Therapy, Journal of Pain and Symptom Management. (February 2020) 59, no. 2, 233–241, 10.1016/j.jpainsymman.2019.10.002. 31610271

[22] Ganz P. A. , Petersen L. , Bower J. E. , and Crespi C. M. , Impact of Adjuvant Endocrine Therapy on Quality of Life and Symptoms: Observational Data Over 12 Months From the Mind-Body Study, Journal of Clinical Oncology. (March 2016) 34, no. 8, 816–824, 10.1200/JCO.2015.64.3866, 2-s2.0-84960414620. 26786934 PMC4872009

[23] Rosenberg S. M. , Stanton A. L. , Petrie K. J. , and Partridge A. H. , Symptoms and Symptom Attribution Among Women on Endocrine Therapy for Breast Cancer, The Oncologist. (June 2015) 20, no. 6, 598–604, 10.1634/theoncologist.2015-0007, 2-s2.0-84932613367. 25933930 PMC4571793

[24] Jing F. , Zhu Z. , Qiu J. , Tang L. , Xu L. , and Xing W. , Symptom Profiles and Related Factors Among Breast Cancer Patients Undergoing Endocrine Therapy: A Latent Profile Analysis, Cancer Nursing. (December 2022) 46, no. 5, E297–E304, 10.1097/NCC.0000000000001125. 37607380

[25] Bodai B. I. and Tuso P. , Breast Cancer Survivorship: A Comprehensive Review of Long-Term Medical Issues and Lifestyle Recommendations, The Permanente Journal. (2015) 19, no. 2, 48–79, 10.7812/TPP/14-241, 2-s2.0-85018214832. PMC4403581 25902343

[26] Miaskowski C. , Barsevick A. , Berger A. et al., Advancing Symptom Science Through Symptom Cluster Research: Expert Panel Proceedings and Recommendations, Journal of the National Cancer Institute. (January 2017) 109, no. 4, 10.1093/jnci/djw253, 2-s2.0-85017932481. PMC5939621 28119347

[27] Lopes-Júnior L. C. , Cancer Symptom Clusters: From the Lab Bench to Clinical Practice, Revista Brasileira de Enfermagem. (October 2022) 75, no. 5, 10.1590/0034-7167-2022v75n5inov. 36287434

[28] Budhwani S. , Moineddin R. , Wodchis W. P. , Zimmermann C. , and Howell D. , Do Longitudinally Collected Symptom Scores Predict Time to Death in Advanced Breast Cancer: A Joint Modeling Analysis, Journal of Pain and Symptom Management. (May 2020) 59, no. 5, 1009–1018, 10.1016/j.jpainsymman.2019.12.006. 31837454

[29] Su Z. , Zhou Y. , Han X. , Pang Y. , He S. , and Tang L. , Symptom Burden in Advanced Breast Cancer Patients and Its Association Between Death Anxiety and Psychological Distress, Chinese Journal of Cancer Research. (June 2022) 34, no. 3, 298–308, 10.21147/j.issn.1000-9604.2022.03.09. 35873892 PMC9273575

[30] Brasil , Instituto Nacional de Câncer José Alencar Gomes da Silva , and Coordenação de Prevenção e Vigilância , Estimativa 2023: Incidência Do Câncer No Brasil, 2022.

[31] Santos G. E. , Sample Calculation: Online Calculator, http://www.calculoamostral.vai.la.

[32] Portenoy R. K. , Thaler H. T. , Kornblith A. B. et al., The Memorial Symptom Assessment Scale: an Instrument for the Evaluation of Symptom Prevalence, Characteristics and Distress, European Journal of Cancer. (1994) 30, no. 9, 1326–1336, 10.1016/0959-8049(94)90182-1, 2-s2.0-0028033880. 7999421

[33] Menezes J. R. d. , Luvisaro B. M. O. , Rodrigues C. F. , Muzi C. D. , and Guimarães R. M. , Test-Retest Reliability of Brazilian Version of Memorial Symptom Assessment Scale for Assessing Symptoms in Cancer Patients, Einstein (São Paulo). (April 2017) 15, no. 2, 148–154, 10.1590/S1679-45082017AO3645, 2-s2.0-85035317255. 28767911 PMC5609609

[34] Daniel W. W. and Cross C. L. , Biostatistics: A Foundation for Analysis in the Health Sciences, 2018, 10th edition, Wiley.

[35] Booth J. G. , Hall P. , and Wood A. T. A. , Balanced Importance Resampling for the Bootstrap, Annals of Statistics. (1993) 21, no. 1, 286–298, 10.1214/aos/1176349026.

[36] Everitt B. , Cluster Analysis, 1974, Heinemann Education Books.

[37] Leisch F. , A Toolbox for k-Centroids Cluster Analysis, Computational Statistics & Data Analysis. (2006) 51, no. 2, 526–544, 10.1016/j.csda.2005.10.006, 2-s2.0-33750289836.

[38] Han C. J. , Reding K. , Cooper B. A. et al., Symptom Clusters in Patients With Gastrointestinal Cancers Using Different Dimensions of the Symptom Experience, Journal of Pain and Symptom Management. (August 2019) 58, no. 2, 224–234, 10.1016/j.jpainsymman.2019.04.035, 2-s2.0-85066260006. 31077784 PMC6679763

[39] Sullivan C. W. , Leutwyler H. , Dunn L. B. et al., Differences in Symptom Clusters Identified Using Symptom Occurrence Rates Versus Severity Ratings in Patients With Breast Cancer Undergoing Chemotherapy, European Journal of Oncology Nursing. (2017) 28, 10.1016/j.ejon.2017.04.001, 2-s2.0-85018733319. PMC5494962 28478849

[40] Baggott C. , Cooper B. A. , Marina N. , Matthay K. K. , and Miaskowski C. , Symptom Cluster Analyses Based on Symptom Occurrence and Severity Ratings Among Pediatric Oncology Patients During Myelosuppressive Chemotherapy, Cancer Nursing. (January 2012) 35, no. 1, 19–28, 10.1097/NCC.0b013e31822909fd, 2-s2.0-84857032989. 21921793 PMC3237960

[41] Suwisith N. , Hanucharurnkul S. , Dodd M. et al., Symptom Clusters and Functional Status of Women With Breast Cancer, Thai Journal of Nursing. (2008) 12.

[42] Phillips K. M. , Pinilla-Ibarz J. , Sotomayor E. et al., Quality of Life Outcomes in Patients With Chronic Myeloid Leukemia Treated With Tyrosine Kinase Inhibitors: A Controlled Comparison, Supportive Care in Cancer. (April 2013) 21, no. 4, 1097–1103, 10.1007/s00520-012-1630-5, 2-s2.0-84879798859. 23179489

[43] Karthikeyan G. , Jumnani D. , Prabhu R. , Manoor U. K. , and Supe S. S. , Prevalence of Fatigue Among Cancer Patients Receiving Various Anticancer Therapies and Its Impact on Quality of Life: A Cross-Sectional Study, Indian Journal of Palliative Care. (September 2012) 18, no. 3, 165–175, 10.4103/0973-1075.105686, 2-s2.0-84873603654. 23439783 PMC3573470

[44] Lawrence D. P. , Kupelnick B. , Miller K. , Devine D. , and Lau J. , Evidence Report on the Occurrence, Assessment, and Treatment of Fatigue in Cancer Patients, Journal of the National Cancer Institute Monographs. (2004) no. 32, 40–50, 10.1093/jncimonographs/lgh027, 2-s2.0-13844262202. 15263040

[45] Bower J. E. , Bak K. , Berger A. et al., Screening, Assessment, and Management of Fatigue in Adult Survivors of Cancer: An American Society of Clinical Oncology Clinical Practice Guideline Adaptation, Journal of Clinical Oncology. (June 2014) 32, no. 17, 1840–1850, 10.1200/JCO.2013.53.4495, 2-s2.0-84903783009. 24733803 PMC4039870

[46] Muthanna F. M. S. , Karuppannan M. , Hassan B. A. R. , and Mohammed A. H. , Impact of Fatigue on Quality of Life Among Breast Cancer Patients Receiving Chemotherapy, Osong Public Health and Research Perspectives. (April 2021) 12, no. 2, 115–125, 10.24171/j.phrp.2021.12.2.09. 33980002 PMC8102880

[47] Williams A. M. , Khan C. P. , Heckler C. E. et al., Fatigue, Anxiety, and Quality of Life in Breast Cancer Patients Compared to Non-Cancer Controls: A Nationwide Longitudinal Analysis, Breast Cancer Research and Treatment. (May 2021) 187, no. 1, 275–285, 10.1007/s10549-020-06067-6. 33392843 PMC8080260

[48] Jacobsen P. B. , Hann D. M. , Azzarello L. M. , Horton J. , Balducci L. , and Lyman G. H. , Fatigue in Women Receiving Adjuvant Chemotherapy for Breast Cancer: Characteristics, Course, and Correlates, Journal of Pain and Symptom Management. (October 1999) 18, no. 4, 233–242, 10.1016/s0885-3924(99)00082-2, 2-s2.0-0032875229. 10534963

[49] Broeckel J. A. , Jacobsen P. B. , Horton J. , Balducci L. , and Lyman G. H. , Characteristics and Correlates of Fatigue After Adjuvant Chemotherapy for Breast Cancer, Journal of Clinical Oncology. (May 1998) 16, no. 5, 1689–1696, 10.1200/JCO.1998.16.5.1689, 2-s2.0-0031803502. 9586880

[50] Seruga B. , Zhang H. , Bernstein L. J. , and Tannock I. F. , Cytokines and Their Relationship to the Symptoms and Outcome of Cancer, Nature Reviews Cancer. (November 2008) 8, no. 11, 887–899, 10.1038/nrc2507, 2-s2.0-54949101585. 18846100

[51] Kim E. , Jahan T. , Aouizerat B. E. et al., Differences in Symptom Clusters Identified Using Occurrence Rates Versus Symptom Severity Ratings in Patients at the End of Radiation Therapy, Cancer Nursing. (November 2009) 32, no. 6, 429–436, 10.1097/NCC.0b013e3181b046ad, 2-s2.0-73649109229. 19816162 PMC2885763

[52] Bower J. E. , Ganz P. A. , Tao M. L. et al., Inflammatory Biomarkers and Fatigue During Radiation Therapy for Breast and Prostate Cancer, Clinical Cancer Research. (September 2009) 15, no. 17, 5534–5540, 10.1158/1078-0432.CCR-08-2584, 2-s2.0-69949108874. 19706826 PMC2884979

[53] Wratten C. , Kilmurray J. , Nash S. et al., Fatigue During Breast Radiotherapy and Its Relationship to Biological Factors, International Journal of Radiation Oncology, Biology, Physics. (May 2004) 59, no. 1, 160–167, 10.1016/j.ijrobp.2003.10.008, 2-s2.0-14644405515. 15093912

[54] Miaskowski C. , Dodd M. , Lee K. et al., Preliminary Evidence of an Association Between a Functional interleukin-6 Polymorphism and Fatigue and Sleep Disturbance in Oncology Patients and Their Family Caregivers, Journal of Pain and Symptom Management. (October 2010) 40, no. 4, 531–544, 10.1016/j.jpainsymman.2009.12.006, 2-s2.0-77957763039. 20570482 PMC2952712

[55] Yates P. , Miaskowski C. , Cataldo J. K. et al., Differences in Composition of Symptom Clusters Between Older and Younger Oncology Patients, Journal of Pain and Symptom Management. (June 2015) 49, no. 6, 1025–1034, 10.1016/j.jpainsymman.2014.11.296, 2-s2.0-84931956584. 25582681

[56] Huang J. , Gu L. , Zhang L. , Lu X. , Zhuang W. , and Yang Y. , Symptom Clusters in Ovarian Cancer Patients With Chemotherapy After Surgery: A Longitudinal Survey, Cancer Nursing. (March 2016) 39, no. 2, 106–116, 10.1097/NCC.0000000000000252, 2-s2.0-84959373900. 25837811

[57] Borniger J. C. , Cancer as a Tool for Preclinical Psychoneuroimmunology, Brain, Behavior, and Immunity. (September 2021) 18, 10.1016/j.bbih.2021.100351. PMC8710415 34988496

[58] Barsevick A. M. and Aktas A. , Cancer Symptom Cluster Research: New Perspectives and Tools, Current Opinion in Supportive and Palliative Care. (March 2013) 7, no. 1, 36–37, 10.1097/SPC.0b013e32835defac, 2-s2.0-84877962459. 23314017

[59] Dantzer R. , Meagher M. W. , and Cleeland C. S. , Translational Approaches to Treatment-Induced Symptoms in Cancer Patients, Nature Reviews Clinical Oncology. (May 2012) 9, no. 7, 414–426, 10.1038/nrclinonc.2012.88, 2-s2.0-84863493750. PMC3412618 22641361

[60] Gilbertson-White S. , Aouizerat B. E. , and Miaskowski C. , Methodologic Issues in the Measurement of Cytokines to Elucidate the Biological Basis for Cancer Symptoms, Biological Research For Nursing. (January 2011) 13, no. 1, 15–24, 10.1177/1099800410379497, 2-s2.0-78650777670. 20798153

[61] Goldszmid R. S. and Trinchieri G. , The Price of Immunity, Nature Immunology. (October 2012) 13, no. 10, 932–938, 10.1038/ni.2422, 2-s2.0-84866528138. 22990891

[62] Yi M. , Li T. , Niu M. et al., Targeting Cytokine and Chemokine Signaling Pathways for Cancer Therapy, Signal Transduction and Targeted Therapy. (July 2024) 9, no. 1, 10.1038/s41392-024-01868-3. PMC11275440 39034318

[63] Silveira D. S. C. , Veronez L. C. , Lopes-Júnior L. C. , Anatriello E. , Brunaldi M. O. , and Pereira-da-Silva G. , Lactobacillus Bulgaricus Inhibits Colitis-Associated Cancer via a Negative Regulation of Intestinal Inflammation in Azoxymethane/Dextran Sodium Sulfate Model, World Journal of Gastroenterology. (November 2020) 26, no. 43, 6782–6794, 10.3748/wjg.v26.i43.6782. 33268961 PMC7684459

[64] Founds S. , Systems Biology for Nursing in the Era of Big Data and Precision Health, Nursing Outlook. (May 2018) 66, no. 3, 283–292, 10.1016/j.outlook.2017.11.006, 2-s2.0-85042089763. 29573828

[65] Broadbent D. E. , Cooper P. F. , FitzGerald P. , and Parkes K. R. , The Cognitive Failures Questionnaire (CFQ) and Its Correlates, British Journal of Clinical Psychology. (February 1982) 21, no. 1, 1–16, 10.1111/j.2044-8260.1982.tb01421.x, 2-s2.0-0019979289. 7126941

[66] Miaskowski C. and Lee K. A. , Pain, Fatigue, and Sleep Disturbances in Oncology Outpatients Receiving Radiation Therapy for Bone Metastasis: A Pilot Study, Journal of Pain and Symptom Management. (May 1999) 17, no. 5, 320–332, 10.1016/s0885-3924(99)00008-1, 2-s2.0-0032914215. 10355211

[67] Gift A. G. , Jablonski A. , Stommel M. , and Given C. W. , Symptom Clusters in Elderly Patients With Lung Cancer, Oncology Nursing Forum. (2004) 31, no. 2, 203–212, 10.1188/04.ONF.203-212. 15017438

[68] Albusoul R. M. , Berger A. M. , Gay C. L. , Janson S. L. , and Lee K. A. , Symptom Clusters Change Over Time in Women Receiving Adjuvant Chemotherapy for Breast Cancer, Journal of Pain and Symptom Management. (May 2017) 53, no. 5, 880–886, 10.1016/j.jpainsymman.2016.12.332, 2-s2.0-85011983747. 28062343 PMC5410185

[69] Marshall S. A. , Yang C. C. , Ping Q. , Zhao M. , Avis N. E. , and Ip E. H. , Symptom Clusters in Women With Breast Cancer: An Analysis of Data From Social Media and a Research Study, Quality of Life Research. (March 2016) 25, no. 3, 547–557, 10.1007/s11136-015-1156-7, 2-s2.0-84958766142. 26476836 PMC5129624

[70] Fu O. S. , Crew K. D. , Jacobson J. S. et al., Ethnicity and Persistent Symptom Burden in Breast Cancer Survivors, Journal of Cancer Survivorship. (December 2009) 3, no. 4, 241–250, 10.1007/s11764-009-0100-7, 2-s2.0-77949885192. 19859813 PMC4852143

[71] Evangelista A. L. and Santos E. M. , Cluster of Symptoms in Women with Breast Cancer Treated with Curative Intent, Supportive Care in Cancer. (July 2012) 20, no. 7, 1499–1506, 10.1007/s00520-011-1238-1, 2-s2.0-84863987277. 21845454

[72] Chow S. , Wan B. A. , Pidduck W. et al., Symptom Clusters in Patients With Breast Cancer Receiving Radiation Therapy, European Journal of Oncology Nursing. (October 2019) 42, 14–20, 10.1016/j.ejon.2019.07.004, 2-s2.0-85069878091. 31446259

[73] Browall M. , Brandberg Y. , Nasic S. et al., A Prospective Exploration of Symptom Burden Clusters in Women With Breast Cancer During Chemotherapy Treatment, Supportive Care in Cancer. (May 2017) 25, no. 5, 1423–1429, 10.1007/s00520-016-3527-1, 2-s2.0-85006138053. 27981366 PMC5378737

[74] Lengacher C. A. , Reich R. R. , Post-White J. et al., Mindfulness Based Stress Reduction in Post-Treatment Breast Cancer Patients: An Examination of Symptoms and Symptom Clusters, Journal of Behavioral Medicine. (2012) 35, no. 1, 86–94, 10.1007/s10865-011-9346-4, 2-s2.0-84858804198. 21506018

[75] So W. K. W. , Law B. M. H. , Ng M. S. N. et al., Symptom Clusters Experienced by Breast Cancer Patients at Various Treatment Stages: A Systematic Review, Cancer Medicine. (April 2021) 10, no. 8, 2531–2565, 10.1002/cam4.3794. 33749151 PMC8026944

[76] Nunes K. Z. , Grippa W. R. , Lopes A. B. et al., Cancer Symptom Clusters, Cardiovascular Risk, and Quality of Life of Patients With Cancer Undergoing Chemotherapy: A Longitudinal Pilot Study, Medicine (Baltimore). (April 2024) 103, no. 16, 10.1097/MD.0000000000037819. PMC11029927 38640317

[77] Silva R. J. G. , Grippa W. R. , Pessanha R. M. , Enriquez-Martinez O. G. , Neto L. C. B. S. , and Lopes-Júnior L. C. , Neutrophil/Lymphocyte Ratio and Platelet/Lymphocyte Ratio and Their Relationship With Nutritional Status and Quality of Life of Hospitalized Women With Breast Cancer, Nutrition and Cancer. (2024) 76, no. 3, 296–304, 10.1080/01635581.2024.2304689. 38287698

[78] Velasco Y. R. J. , Carvalho Fernandes A. F. , de Freitas C. E. , Moura Barbosa Castro R. C. , Sixsmith J. , and Lopes-Júnior L. C. , Palliative Care in the Treatment of Women With Breast Cancer: A Scoping Review, Palliative & Supportive Care. (June 2024) 22, no. 3, 592–609, 10.1017/S1478951523001840. 38058195

[79] Lopes-Júnior L. C. , Grassi J. , Freitas M. B. et al., Cancer Symptom Clusters in Children and Adolescents With Cancer Undergoing Chemotherapy: A Systematic Review, Nursing Reports. (May 2025) 15, no. 5, 10.3390/nursrep15050163. PMC12114543 40423197

[80] Silva R. J. G. , Grippa W. R. , Pessanha R. M. et al., Cancer Symptom Cluster in Hospitalized Women With Breast Cancer: A Descriptive Observational Study, Revista Brasileira de Enfermagem. (April 2025) 78, no. 2, 10.1590/0034-7167-2024-0091. PMC12037190 40298695

[81] Harrington L. , Precision Nursing, AACN Advanced Critical Care. (September 2021) 32, no. 3, 243–246, 10.4037/aacnacc2021471. 34490440

[82] Lopes-Júnior-Lc , Precision Nursing: Advances and Challenges in Implementation, Revista Latino-Americana de Enfermagem. (2025) 33, 10.1590/1518-8345.8046.4684. PMC12562620 41172388

[83] Veronez L. C. , Silveira D. S. C. D. , Lopes-Júnior L. C. , Dos Santos J. C. , Barbisan L. F. , and Pereira-da-Silva G. , Jacalin Attenuates Colitis-Associated Colorectal Carcinogenesis by Inhibiting Tumor Cell Proliferation and Intestinal Inflammation, Inflammatory Bowel Diseases. (May 2025) 31, no. 5, 1344–1354, 10.1093/ibd/izae303. 39745886

